# Supplementary material for: DNA damage checkpoint activation impairs chromatin homeostasis and promotes mitotic catastrophe during aging
Source: eLife. 2019 Nov 12;8:e50778. doi: 10.7554/eLife.50778 (PMC6850777; doi:10.7554/eLife.50778)
Supplement: Supplementary file 1. [file elife-50778-supp1.docx]

| **Strain Name** | **Genotype** | **Source** |
| --- | --- | --- |
| BY4741 | MATa his3∆1 leu2∆0 met15∆0 ura3∆0 |  |
| BY4742 | MATα his3∆1 leu2∆0 lys2∆0 ura3∆0 |  |
| YSI129 | ade2-1 ura3-1 his3-11 trp1-1 leu2-3,112 can1-100 fob1∆::LEU2 his3-11::GFP-LacI-HIS3 LacO(50)-ADE2:445kb ChXII (110 rDNA copies) | Ide, et al. 2010 |
| AMY914 | MATa ura3 trp1 his3-11 MET-CDC20::URA pURA::tetR-GFP::LEU2 cenIV::tetOx448::URA3 | Fernius, et al. 2009 |
| AMY1081 | MATa ade2-1 leu2-3 trp1-1 trp1 his3-11 can1-100 GAL psi+ MET-CDC20::URA pURA::tetR-GFP::LEU2 ura3::tetOx112::URA3 | Fernius, et al. 2009 |
| MC213 | HTB2::mCherry-URA3 his3 leu2 met15 LYS2 | this study |
| MC230 | fob1∆::KanMX his3 leu2 HTB2::mCherry-URA met15 LYS2 | this study |
| MC237 | HTA2::GFP-HIS3 his3 leu2 ura3 met15 LYS2 | Yeast GFP Collection |
| MC239 | HTB2::GFP-HIS3 his3 leu2 ura3 met15 LYS2 | Yeast GFP Collection |
| MC245 | NUP49::GFP-HIS3 his3 leu2 ura3 met15 LYS2 | Yeast GFP Collection |
| MC247 | TUB1::GFP-HIS3 his3 leu2 ura3 met15 LYS2 | Yeast GFP Collection |
| MC250 | HTB2::mCherry-URA3 WHI5::GFP-HIS3 leu2 met15 LYS2 | this study |
| MC255 | spt21∆::KanMX his3 leu2 met15 LYS2 | Yeast KO Collection |
| MC257 | hpc2∆::KanMX his3 leu2 met15 LYS2 | Yeast KO Collection |
| MC258 | NUP49::GFP-HIS3 his3 leu2 ura3 met15 LYS2 htb2::mCherry-URA | this study |
| MC263 | HTB2::mCherry-URA3 his3 leu2 met15 lys2 hpc2∆::KanMX | this study |
| MC264 | HTB2::mCherry-URA3 TUB1::GFP-HIS2 leu2 met15 LYS2 | this study |
| MC266 | HTB2::mCherry-URA3 his2 leu2 MET15 lys2 spt21∆::KanMX | this study |
| MC273 | rad52∆:KanMX HTB2:mCherry-URA his3 leu2 ura3 met15 LYS2 | this study |
| MC281 | HTB2::mCherry-URA3 his3-11::GFP-LacI-HIS3 RDN1::LacO(50)-ADE2 | this study |
| MC285 | HTB2::mCherry-URA3 his3-11::GFP-LacI-HIS3 LacO(50)-ADE2:445kb ChXII | this study |
| MC349 | mad3∆::KanMX his3 leu2 htb2::mCherry-URA met15 LYS2 | this study |
| MC351 | SPC72::GFP-HIS3 his3 leu2 ura3 met15 LYS2 | Yeast GFP Collection |
| MC352 | UTP13::GFP-HIS3 his3 leu2 ura3 met15 LYS2 | Yeast GFP Collection |
| MC354 | CDC14::GFP-HIS3 his3 leu2 ura3 met15 LYS2 | Yeast GFP Collection |
| MC355 | SPC72::GFP-HIS3 his3 leu2 ura3 met15 LYS2 HTB2:mCherry-URA | this study |
| MC360 | CDC14::GFP-HIS3 his3 leu2 ura3 met15 LYS2 HTB2:mCherry-URA | this study |
| MC364 | UTP13::GFP-HIS3 his3 leu2 ura3 met15 LYS2 HTB2:mCherry-URA | this study |
| MC367 | mad3∆::KanMX his3 leu2 ura3 met15 LYS2 HTB2:mCherry-URA | this study |
| MC372 | tom1∆::KanMX his3 leu2 ura3 met15 LYS2 HTB2:mCherry-URA | this study |
| MC394 | ura3 his3-11 pURA::tetR-GFP::LEU2 cenIV::tetOx448::URA3 HTB2:mCherry-URA | this study |
| MC395 | leu2-3 his3-11 pURA::tetR-GFP::LEU2 ura3::tetOx112::URA3 HTB2:mCherry-URA | this study |
| MC516 | bfa1∆:KanMX HTB2:mCherry-URA his3 leu2 ura3 met15 LYS2 | this study |
| MC523 | ies4∆::KanMX his3 leu2 MET15 lys2 | Yeast KO Collection |
| MC532 | rad9∆:KanMX HTB2:mCherry-URA his3 leu2 ura3 met15 LYS2 | this study |
| MC535 | ies4∆::KanMX HTB2:mCherry-URA his3 leu2 ura3 met15 LYS2 | this study |
| GS408 | tom1∆::KanMX his3 leu2 MET15 lys2 | Yeast KO Collection |
