# Supplementary material for: DNA damage checkpoint activation impairs chromatin homeostasis and promotes mitotic catastrophe during aging
Source: eLife. 2019 Nov 12;8:e50778. doi: 10.7554/eLife.50778 (PMC6850777; doi:10.7554/eLife.50778)
Supplement: Supplementary file 2. [file elife-50778-supp2.docx]

| **Strain Name** | **Background** | Method | Uncensored RLS **(Median)** | Censored RLS **(Median)** |
| --- | --- | --- | --- | --- |
| MC237 | Hta2:GFP | µFluidic | 18 | 23 |
| MC239 | Htb2:GFP | µFluidic | 18 | 21 |
| MC213 | Htb2:mCherry | µFluidic | 15 | 17 |
| MC230 | Htb2:mCherry fob1∆ | µFluidic | 20 | 22 |
| MC532 | Htb2:mCherry *rad9*∆ | µFluidic | 11 | 12 |
| MC273 | Htb2:mCherry *rad52*∆ | µFluidic | 8 | 14 |
| MC535 | Htb2:mCherry *ies4*∆ | µFluidic | 14 | 16 |
| MC372 | Htb2:mCherry *tom1*∆ | µFluidic | 18 | 21 |
| MC263 | Htb2:mCherry *hpc2*∆ | µFluidic | 18 | 25 |
| MC266 | Htb2:mCherry *spt21*∆ | µFluidic | 15 | 18 |
| MC516 | Htb2:mCherry *bfa1*∆ | µFluidic | 9 | 12 |
| BY4741 | Wildtype | microdissection | 25 | 25 |
| GS408 | *tom1*∆ | microdissection | 29 | 29 |
| MC523 | *ies4*∆ | microdissection | 31 | 31 |
